# A Survey-Based Investigation of Human Factors Associated With Transport Related Injuries in Horses

**DOI:** 10.3389/fvets.2018.00294

**Published:** 2018-11-22

**Authors:** Barbara Padalino, Chris W. Rogers, Danielle Guiver, Kirrilly R. Thompson, Christopher B. Riley

**Affiliations:** ^1^Jockey Club College of Veterinary Medicine and Life Sciences, City University of Hong Kong, Kowloon, Hong Kong; ^2^Department of Veterinary Medicine, University of Bari, Bari, Italy; ^3^School of Veterinary Science, Massey University, Palmerston North, New Zealand; ^4^UniSA Business School, University of South Australia, Adelaide, SA, Australia

**Keywords:** transport, injury, horse, human factors, experience

## Abstract

Injuries resulting from road transport are common in horses and are a potential welfare concern, as well as, a source of economic loss. An online cross sectional survey was used to determine the prevalence of road transport related injuries to horses in New Zealand and the association of human factors including demographics, industry background, training and the horse handling experience of the respondents with transport related injury. The survey generated 1133 valid responses that were analyzed using descriptive statistics, univariate and multivariate logistic regression analysis. At least one injured horse was reported by 201/1133 (17.7%) respondents as occurring during the two previous years. Only 191 respondents chose to provide further information on when the injury occurred and most injuries (133/191; 69.6%) occurred in transit. The respondent perceived possible reason for injury was reported by 190, and was most frequently thought to be either horse-associated (87/190; 45.8%) or associated with a driver mistake (18/190; 9.5%). Variables that remained as significantly associated with injury in a multivariate model focusing on human factors were experience in horse handling, the industry sector, and the amateur or professional involvement with the horse industry. The odds of injury associated with professionals may reflect greater exposure due to more frequent transport and larger numbers of horses in their care than amateurs. Findings confirm that human factors are associated with the risk of an injury during transport. Although further studies are required to determine if any of these relationships are causative, education on transport best practices with consideration of these factors may mitigate their influence.

## Introduction

Traumatic injuries are a common cause of morbidity and mortality in horses (1–4). These are not only a welfare concern, but also a significant cause of economic loss for the equine industry (3, 4). Factors that have been reported as associated with an increase of traumatic injury include a range of management practices, horse signalment, type of use, and horse transport (2, 5–7). In Sweden horse trailers (i.e., floats in Australasia) have been identified as the second most common location after the paddock where injury to horses occurs (8).

The incidence of transport-related horse injuries has been most extensively studied for horses transported by road using commercial companies and varies from 1.6 to 33% depending upon the population studied (9–11). In Australia injuries associated with commercial and non-commercial equine transport were reported by 45% of surveyed respondents within a 2-year period prior to survey completion (12). Human factors including the age of the respondents (younger vs. older), the choice to use protective equipment on the horse and administration of sedation have been identified as risk factors for injury (12). In a Swedish survey of equestrian horse owners 12% reported an equine injury during loading, and 5% described concurrent injury of the animal being loaded and the handler (13), but risk analysis was not performed. A face-to-face survey conducted at equestrian events in Southern Australia that focused on non-commercial horse transport, reported that 25% of the respondents had experienced a transported related injury within the 15 years prior to the study (14). In the latter study, the age and the experience of the respondents were not identified as risk factors. However, 6% of the respondents admitted that the cause of the transport-related injury they recalled was a driver error, and respondents who admitted answering the telephone while driving had an increased likelihood of having previously had a horse injured during transport.

Driver skills and behaviors are of critical importance with respect to safe live animal transportation because they affect the movement of the vehicle and therefore the ability of the animals to balance during movement (15). Driver experience has been postulated as a human related risk factor and although equine-related data is currently lacking, the probability of animals becoming non-ambulatory or markedly lame during transport is greater for drivers hauling cattle (for example) with >6 years of experience than those that are more experienced (16). Despite recent advancements in the understanding of the risk of transport related injuries to horses during commercial and non-commercial movement, information on human related factors associated with these injuries are limited, as are strategies that mitigate the risk.

In New Zealand the horse population is estimated at 99,000 (17), and the number of horse owners and equine industry workers at approximately 90,000 people (17–19). This figure encompasses amateur and professional respondents, racing, breeding, equestrian sports and recreational activities. Differences in the patterns of horse movements have been reported between the racing, breeding (20, 21) and non-racing sectors in New Zealand (22). The extrapolation of these findings to the national equine population (17) results in a conservative estimate of four million horse movements per year in New Zealand, indicating that the exposure to transport related injury may be significant. With respect to the road transport of horses, information on the industry-wide demographics of the horse owners and industry workers, their equine industry related education and training background, and driver behaviors in New Zealand is limited. In New Zealand the relationship between these and other human factors with respect to injuries sustained by horses during their road transport in non-commercially operated vehicles has not been evaluated. The aim of this work was to investigate whether the demographics, industry related background, training and experience of the respondents were associated with the odds of injury to horses during road transport in New Zealand.

## Materials and methods

This online survey was approved by the Massey University Human Ethics Committee as low risk (Ethics Notification Number: 4000017178).

### Respondents

The target population for this survey were residents of New Zealand associated with the road transport of horses for professional or recreational purposes. Respondents were required to have at least one horse in their care and to have organized transport for horse(s) at least once during the 2 years prior to completing the survey. Based on an estimated target population of 90,000 equine industry participants (18, 19) 1,055 surveys were required to attain a 95% confidence level, and an error level of ± 3% (23).

### Survey

As part of a wider study of horse transport in New Zealand, the human factors section of this survey (Supplementary Material) consisted of 17 closed and 8 open ended questions that sought to elicit from respondents their demographic details (gender, age), information on their involvement with the equine industry (sector, membership with one or more New Zealand based horse organizations, the nature of their involvement with horses (professional or amateur) and the specific type of activities in which they participated), their equine industry related experience, education and training (experience in horse handling, industry qualifications, class of driving license, understanding of the Animal Welfare Code (AWC) (24), their ability to recognize a horse in distress, and the frequency of performing an assessment of fitness for travel, the use of a training for loading and traveling), their horse and journey details (number of horses in their care, travel frequency and distances transported). In addition to soliciting the information described above, survey respondents were asked to respond to questions that described the most recent transport-related injury (a shallow or deep cut or wound, fracture or broken bone, bruise, skin or tail rubbed raw, or other), if any, that had occurred to a horse during the 2 years prior to them completing the survey (2015–2017).

The survey was developed by a process of iterative review by the researchers taking into account the key design features required to ensure valid questionnaire results (25, 26). The survey was constructed using online proprietary software (Qualtrics, New Zealand), piloted among six horse owning staff members at Massey University, and adjustments made in response to feedback. Invitations to participate and to distribute or access the link to the survey were disseminated via horse organization office bearers or their members (initially contacted by one of the researchers (CBR) by telephone with a reminder call 6 weeks after the survey was opened). These contacts forwarded the survey link to members and industry contacts via email, web and social networking sites such as Facebook pages (Table S1). The survey link was further shared by supportive individuals in a social media version of “snowball sampling” (27, 28). Proprietors of two businesses and one academic institution disseminated the survey link online to their clients and staff, respectively, via email, web and social media pages. The survey link was available for completion between 7 February 2017 and 16 May 2017 (~3 months).

### Explanatory variables

Quantitative data not fulfilling the requirements for parametric analyses (age, experience, number of horses) were transformed to categorical variables for further analysis, while ensuring a balanced dataset (i.e., no category with >5% of the population of values). For the same reason, respondents from the Thoroughbred and Standardbred racing industry sectors were combined into the same category (variables: industry sector and membership), and respondents with a learner or restricted driving licenses (i.e., without a “full” license) were combined into the same group for analyses. Respondents with one or more equine related qualifications and those without were dichotomised into “qualified” (yes) or “unqualified” categories (no), respectively (Table 1).

**Table 1:** Name and description of the candidate explanatory variables evaluated.

**Name**	**Description**	**Categories**
**RESPONDENTS DETAILS**		
Gender		Male, Female
Age		16–30, 31–45, 46–60, 61+
**INVOLVEMENT WITH THE EQUINE INDUSTRY**		
Industry sector	In which sector of the horse industry were they involved	Thoroughbred (TB) or Standardbred (SB) racing, Dressage, Eventing, Show Jumping, Pony Club, Endurance and Competition Trail Riding (CTR), Horse breeding, Recreational non-competitive, Other (i.e., Hunting, Western, Polo, Showing)
Membership	In what organization did the respondent have a membership	New Zealand Thoroughbred or Harness Racing (Racing), Equestrian Sport New Zealand (ESNZ), New Zealand Pony Club Association (NZPCA), no membership
Involvement	Nature of the respondent's involvement with horses	Professional (involved with horses for financial reward), Amateur (involved with horses as a hobby)
**EXPERIENCE/EDUCATION/TRAINING**		
Experience	Respondent's years of experience handling horses	1–5, 6–10, 11–20, 21–30, 31–40, 41–50, 51+
Qualification	Possession of one or more equine industry qualifications	Yes, No
Driving License	Respondent's class of driving license	Learner or Restricted, Full, Heavy vehicle license (HVL)
AWC	Respondent's self-assessment of their understanding of the Animal Welfare Code (Transport within New Zealand 2011)	1-low, 2-some, 3-moderate, 4-high, 5-very high
Distress	Respondent's self-assessment of ability to identify a horse in distress	1-low, 2-some, 3-moderate, 4-high, 5-very high
FFT	Frequency of assessment of fitness for travel before moving horses	1-never, 2-sometimes, 3-about half the time, 4-most of the time, 5-always
Mechanical check list	Frequency of use of a mechanical check list on the transport vehicle before moving horses	1-never, 2-sometimes, 3-about half the time, 4-most of the time, 5-always
Horse training	Did the respondents train their horse for loading and traveling?	Yes, No
**HORSE AND JOURNEY DETAILS**		
Number of horses	Number of horses kept with their horse	1–2, 3–5, 6–10, 11–15, 16–30, 31+
Journey frequency	Frequency of organized transport events	Daily, 2–5 times a week, once a week, fortnightly, monthly, < once a month
Journey distance	Average journey distance (km)	1–20, 31–60, 61–90, 91–120, 120–240, 241+

### Outcome variable

Injury of a horse in association with transport (binary: no injury; at least one injury) was the outcome for univariate and multivariate logistic regressions. An injury was considered to have been reported by the respondent if they checked the question asking if their horse had sustained a shallow or deep cut or wound, fracture or broken bone, bruise, skin or tail rubbed raw, or other as described by respondents. The respondents reporting at least one injury also provided the following information: when during transport they thought that the injury had occurred, and their role(s) (driver, passenger, person loading and person unloading the horse).

### Statistical analyses

Quantitative data (survey completion time, age, experience, number of horses) were examined using simple descriptive statistics and were plotted and tested for normality using Shapiro-Wilk tests (Statistica 11, Statsoft, Tulsa, OK, USA). Data for these variables were not normally distributed and are descriptively reported as median and interquartile ranges (IQR; Q1–Q3). Descriptive statistics of categorical data were obtained using Statulor^β^ and are reported as the number of responses and percentages (including the number of values of used for analysis, and the number of missing values). Associations between the predictive variables were explored using χ2 tests (significance at *P* < 0.05). A univariate logistic regression models were developed with injury as a binary outcome (injury/non-injury); *P*-values were calculated using the Wald test. Each predictor variable returning a *P* < 0.25 from the univariate analyses was considered for inclusion in the final multivariate model for injury (Table S2). To avoid errors asscociated with collinearity, the variable which was most frequently collinear with the other variables was excluded. Predictor variables for the final multivariate logistic regression model were selected using a step-wise backward elimination procedure whereby predictive variables were removed until all variables in the final model had a *P* < 0.05 indicating significance (12). The findings are presented as odds ratios (OR) and confidence intervals (95% CI) for each predictive variable value. Wald test *P* values were reported for each association. All univariate and multivariate analyses were performed using GenStat^®^Version 14 (VSN International, Hemel Hempstead, UK).

## Results

### Survey response

The survey resulted in 1,486 initial contacts, of which eight did not consent to further participation, 153 (10.3%) consented to participation but did not complete any other questions, 185 (12.5%) did not respond to the question on whether a horse had sustained an injury or not, and 7 (0.5%) did not transport their horses. After these responses were excluded, 1,133 (76.7%) valid responses remained for analysis. This sample size resulted in a 95% confidence level, and an error level of ± 2.9% for the study population. Respondents took approximately 10 min to complete the survey (median: 10, min; IQR 7.6–13.7 min).

### Descriptive statistics of the quantitative and categorical data

The median of age of respondents was 45 years (IQR 31–54 years) and the median duration of experience was 30 years (IQR 15–40 years). The respondents took care of 14,573 horses, with a median of 6 horses (IQR 3–14) per respondent, and the median distance of their road journeys with horses was 50 km (IQR 30–100).

**Figure 1:**
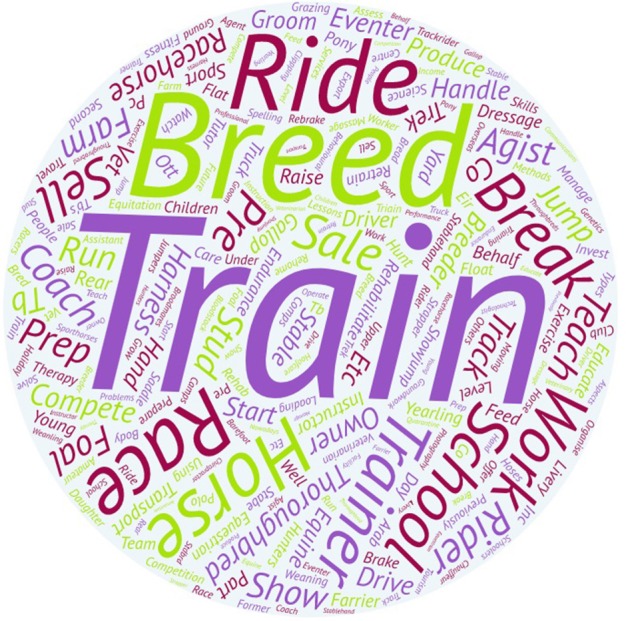
Word art with the description of the professional activities carried out by the professional respondents. The size of a particular word represents a frequency of occurrence within the free text responses of the survey participants. In this case, respondents associated horse transport most frequently with the word “train,” followed by “breed “and “ride.” These words reflect the activities that those professionally engaged in the New Zealand equine industry are most likely to involved with.

**Figure 2:**
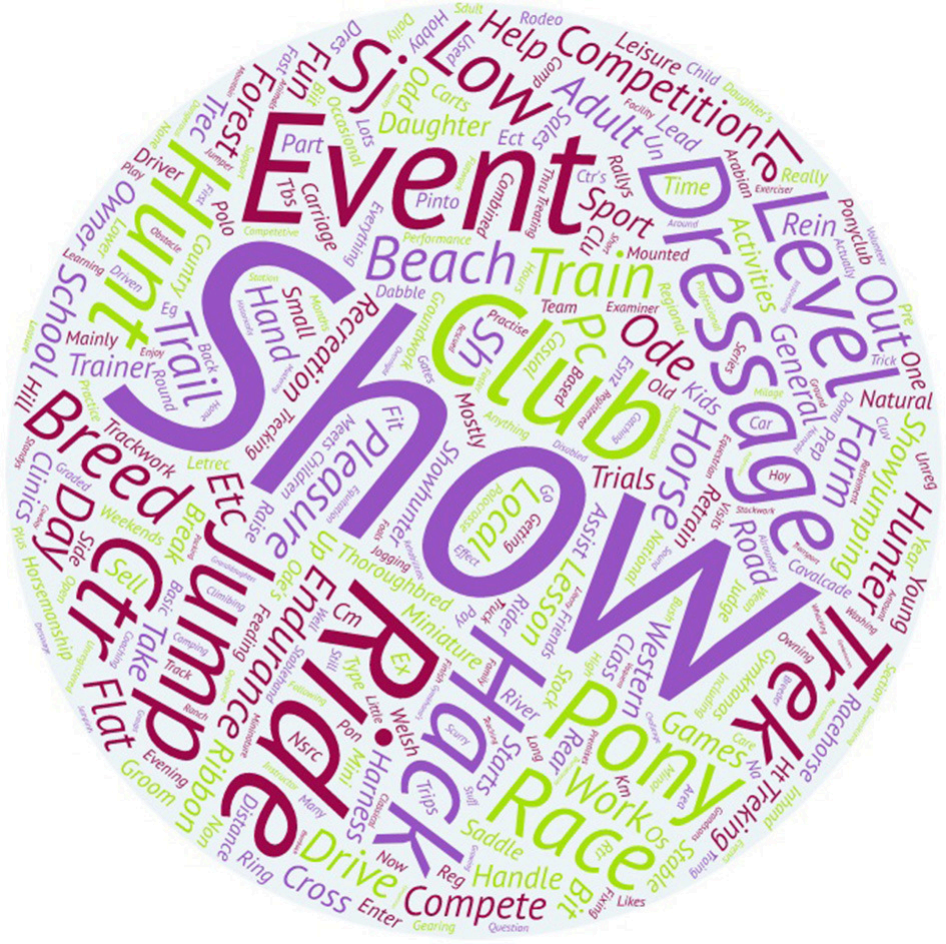
Word art with the description of the amateur activities carried out by the amateur respondents. The size of a particular word represents a frequency of occurrence within the free text responses of the survey participants. In this case, respondents associated horse transport most frequently with the word “show,” followed by “ride” and “event.” “Event” was found to be a significant quantitative contributor to the odds of injury in horses during road transport.

Table 2 shows counts and the percentage breakdown of response classes within the study variables that were surveyed. The description of the type of professional and amateur activities most frequently listed by respondents in free text areas of the survey are shown in Figures 1, 2, respectively.

**Table 2:** Frequency table for demographics characteristics and equine industry involvement of respondents to a survey on horse transportation practices in New Zealand.

**Predictive variable**	**Class**	**Count**	**Percentage**
Gender	Female	947	84.4
	Male	175	15.6
	Total	1,122	100
	Missing values	11	0.9
Age (years)	16–30	276	24.4
	31–45	306	27.1
	46–60	387	34.2
	61+	161	14.3
	Total	1,130	100
	Missing values	3	0.3
Industry Sector	Dressage	131	11.6
	Endurance & Competitive trail riding	54	4.8
	Eventing	120	10.6
	Horse breeding	67	5.9
	Other (Hunting, Western, Polo, Showing, etc.)	96	8.5
	Pony club	78	6.9
	Racing (Thoroughbred and Standardbred)	244	21.5
	Recreational riding	209	18.4
	Show jumping	134	11.8
	Total	1,133	100
Membership	Equestrian Sports New Zealand	423	37.3
	Racing membership	241	21.3
	New Zealand Pony Club Association	98	8.6
	Other (please specify)	171	15.1
	Not registered member of any horse related organization	200	17.7
	Total	1,133	100
Involvement	Amateur	851	75.1
	Professional	282	24.9
	Total	1,133	100
Experience in horse handling (years)	1_5	47	4.2
	6_10	95	8.5
	11_20	271	24.2
	21_30	229	20.4
	31_40	260	23.2
	41_50	149	13.3
	51+	69	6.2
	Total	1,120	100
	Missing values	13	1.1
Equine qualification?	No	446	39.4
	Yes	687	60.6
	Total	1133	100
Number of horses	1_2	197	17.6
	3_5	358	32.1
	6_10	232	20.8
	11_15	96	8.6
	16_30	133	11.9
	31+	100	9.0
	Total	1,116	100
	Missing values	17	1.5
Driving license class	Learner or restricted (probation)	70	6.3
	Full	660	59.4
	Heavy vehicle license	382	34.3
	Total	1,112	100
	Missing values	21	1.8
Understanding of the Animal Welfare Code (Transport within New Zealand 2011)	1–low	198	17.5
	2–some	171	15.1
	3–moderate	394	34.9
	4–high	249	22.1
	5–very high	117	10.4
	Total	1,129	100
	Missing values	4	0.353045
Respondent's self-assessment of ability to identify a horse in distress	1–low	0	0
	2–some	11	1.0
	3–moderate	65	5.7
	4–high	464	41.1
	5–very high	590	52.2
	Total	1,130	100
	Missing values	3	0.3
Assessment of fitness for travel before moving horses	1–never	17	1.5
	2–sometimes	68	6.1
	3–about half the time	50	4.4
	4–most of the time	263	23.3
	5–always	731	64.7
	Total	1,129	100
	Missing values	4	0.3
Mechanical checklist?	1–never	88	7.9
	2–sometimes	204	18.4
	3–about half the time	120	10.8
	4–most of the time	373	33.7
	5–always	323	29.2
	Total	1,108	100
	Missing values	25	2.2
Did the respondents train their horse for loading and traveling?	No	240	21.2
	Yes	893	78.8
	Total	1,133	100
Journey frequency	Daily	79	7.0
	2 to 5 times a week	282	24.9
	Once weekly	276	24.3
	Fortnightly	211	18.6
	Monthly	130	11.5
	Less than once a month	155	13.7
	Total	1,133	100
Journey distance (km)	1_30	315	28.9
	31_60	288	26.4
	61_90	81	7.4
	91_120	168	15.4
	120_240	141	12.9
	241+	98	9.0
	Total	1,091	100
	Missing values	42	3.7

*^1^Of the qualifications documented, the most commonly reported were certificates from the New Zealand Pony Club (58.2%), New Zealand Racing (e.g., licensed trainer, driver, stable hand)(14.3%), Equestrian Sport New Zealand (e.g., Coach, FEI official, Show Hunter Judge)(6.8%)*.

### Descriptive statistic of data related to injury

At least one horse was reported as injured in association with transport by 201/1,133 (17.7%) of respondents. Table 3 shows counts and the percentage breakdown of response on when and why injuries were thought to have occurred, and the respondent's role in the transport injury event described. The majority of the injuries were thought by respondents to have occurred while traveling due to a problem with the horse. Few respondents declared that a driver error was the cause, and the remainder indicated that a mechanical problem, vehicle collision, poor road conditions or a combination of factors had led to the injury. The majority of the respondents reported that when the injury occurred they were acting as either the driver or the person loading/unloading the horse. Some respondents described multiple injuries to their horses, with a total of 287 injuries reported. The most common injuries were mild [shallow cuts (118/287, 41.1%), bruises (78/287, 24.7%) and skin erosions (40/287, 13.9%)], and only a few were reported as sustaining a severe deep cut (46/287, 16.1%) or fractures (12/287, 4.2%).

**Table 3:** Frequency table for injury related questions of respondents (*n* = 201) who affirmed having had at least one horse injured during a previous 2 years before completing a survey on horse transportation practices in New Zealand.

**Question**	**Response**	**Count**	**Percentage**
When during the trip did the injury occur?	Pre-loading	1	0.5
	Loading	19	10.0
	Traveling	133	69.6
	Unloading	12	6.3
	I don't know	26	13.6
	Total	191	
	Missing values	10	4.9
How do you think the injury occurred?	A mistake by the driver	18	9.5
	A problem with the horse	87	45.8
	Poor road condition	4	2.1
	Mechanical problem with the transport vehicle	5	2.6
	Vehicle collision	7	3.6
	Other	69	36.4
	Total	190	
	Missing Value	11	5.5
What was your involvement with the transportation pf the injured horses? (tick all that apply)	Driver	93	53.8
	Passenger	48	27.7
	Person loading	57	32.9
	Person unloading the horse	71	41.0
	Total	173	
	Missing values	28	13.9

### Univariate and multivariate logistic regression

The results of the variables from the univariate logistic regression analyses that were found to have a significant association with injury are reported in Table 4. Younger people (<30 years old) were five times more likely to report a transport related injury than the reference group of older people (>61 years old). Respondents without a full driving license and those who did not always perform a mechanical checklist were also more likely to have reported an experience with a horse injury during transport. Finally, horses managed by respondents with >5 years of experience in horse handling and those using horses for eventing were at the highest risk ofreporting a horse being injured during transport.

**Table 4:** Results of univariate logistic regression analyses of associations between injury and the explanatory variables age, industry sector, experience, driving license class and performance of a mechanical checklist.

**Variable**	**Category**	**Injuries-No *n* (%)**	**Injuries-Yes n (%)**	**OR**	**95%CI**	***P*^a^**
Age (years)	61 +	150 (13.3)	11 (1.0)	Ref		< 0.001
	46–60	335 (29.6)	52 (4.6)	2.11	1.07–4.17	
	31–45	244 (21.6)	62 (5.5)	3.46	1.76–6.78	
	16–30	201 (17.8)	75 (6.6)	5.09	2.61–9.91	
Industry sector	Recreational	176 (15.5)	33 (2.9)	Ref		0.019
	Endurance and competitive trail riding	44 (3.9)	10 (0.9)	1.21	0.55–2.64	
	Dressage	111 (9.8)	20 (1.8)	0.96	0.52–1.75	
	Eventing	83 (7.3)	37 (3.3)	2.38	1.39–4.06	
	Show jumping	108 (9.5)	26 (1.8)	1.28	0.72–2.26	
	Pony club	63 (5.6)	15 (1.3)	1.27	0.64–2.49	
	Horse breeding	59 (5.2)	8 (0.7)	0.72	0.31–1.65	
	Racing	209 (18.4)	35 (3.1)	0.89	0.53–1.49	
	Other	79 (7.0)	17 (1.5)	1.14	0.60–2.18	
Experience in horse handling (years)	51+	64 (5.7)	5 (0.4)	Ref		0.005
	41–50	132 (11.7)	17 (1.5)	1.65	0.58–4.66	
	31–40	219 (19.4)	51 (4.5)	2.39	0.90–6.31	
	21–30	186 (16.5)	43 (3.8)	2.95	1.12–7.79	
	11–20	213 (18.8)	58 (5.1)	3.48	1.34–9.05	
	6–10	75 (6.6)	20 (1.8)	3.41	1.21–9.60	
	1–5	32 (2.8)	15 (1.3)	6.00	2.00–17.96	
Driving license class	HVL^1^	316 (28.4)	66 (5.9)	Ref		0.045
	Full	551 (49.6)	109 (9.8)	1.06	0.75–1.47	
	L+R^2^	50 (4.5)	20 (1.8)	2.02	1.15–3.52	
Mechanical checklist	5–always	268 (24.2)	55 (5.0)	Ref		0.048
	4–most of the time	322 (29.1)	51 (4.6)	0.77	0.51–1.16	
	3–about half the time	97 (8.8)	23 (2.1)	1.15	0.67–1.98	
	2–sometimes	156 (14.1)	48 (4.3)	1.49	0.97–2.31	
	1–never	70 (6.3)	18 (1.6)	1.25	0.69–2.26	

*Data were collected from an online survey on horse transport in New Zealand (n = 1,133) for movements occurring between 2015 and 2017*.

a *P, Wald test P value*.

1 Heavy vehicle license

2 *Learners or restricted (probationary) licenses*.

There was a strong collinearity between age and horse handling experience (χ^2^ = 985.9, *df* = 18, *P* < 0.001), driving license class (χ^2^ = 241.6, *df* = 6, *P* < 0.001), assessment of horse fitness for transport (χ^2^ = 75.86, *df* = 12, *P* < 0.001), and industry sector (χ^2^ = 75.86, df = 24, *P* < 0.001). Age was excluded by the final model to avoid multicollinearity and because it was not a human factor variable under the control of respondents.

The results of the other variables in the univariate regression analyses that were evaluated for inclusion in the multivariate logistic regression model are reported in Table S3. The results of the multivariate logistic regression analysis (χ^2^ = 46.79, *df* = 15 11, *P* < 0.001) are reported in Table 5. The odds of injuries was lower for respondents with more than 5 years of experience in horse handling. Respondents involved in eventing or with horses professionally were at higher odds of reporting a traumatic injury when they moved their horses by road.

**Table 5:** Results of multivariate regression analysis of associations between injury and the explanatory variables experience, industry sector, and type of involvement (amateur/professional).

**Variable**	**Category**	**Estimate**	**SE**	**OR**	**95%CI**	***P*^a^**
Experience in horse handling (years)	51+	Ref		Ref		0.024
	41–50	0.39	0.53	1.48	0.52–4.26	
	31–40	0.78	0.50	2.19	0.82–5.87	
	21–30	0.99	0.50	2.69	1.00–7.23	
	11–20	1.05	0.50	2.87	1.07–7.67	
	6–10	1.02	0.54	2.77	0.96–8.01	
	1–5	1.71	0.57	5.52	1.79–17.05	
Industry sector	Recreational	Ref		Ref		0.024
	Endurance and competitive trail riding	0.34	0.40	1.41	0.63–3.12	
	Dressage	0.80	0.31	1.08	0.58–2.00	
	Eventing	0.87	0.28	2.40	1.38–4.17	
	Show jumping	0.17	0.29	1.19	0.66–2.14	
	Pony club	0.25	0.35	1.28	0.65–2.55	
	Horse breeding	−0.32	0.44	0.72	0.30–1.72	
	Racing	−0.18	0.30	0.83	0.45–1.52	
	Other	0.59	0.33	1.15	0.59–2.21	
Involvement	Amateur	Ref		Ref		0.004
	Professional	0.59	0.20	1.81	1.21–2.71	

*Data were collected from an online survey on horse transport in New Zealand (n = 1,133) for movements occurring between 2015 and 2017. P^a^, Wald test P-value*.

## Discussion

This study investigated whether human factors such as demographics, equine industry background, education and experience of the respondents were associated with transport related injury in horses. In contrast to recent Australian and Swedish studies (the latter focused only on loading injuries) (13, 14), the high number of responses obtained in the current survey ensured a statistically representative sample of the population under study.

The New Zealand equine industry has characteristics that were reflected in the participating respondents. In agreement with the findings of other studies of the New Zealand equine industry, the majority of respondents were mature women, involved in non-racing sectors for amateur purposes for a number of years, moving horses often over short distances, owning or caring for >6 horses (21, 29–31). However, our data provides previously unpublished information on the broader New Zealand equine industry related to their industry relevant educational backgrounds and the nature of their involvement with the industry. The respondents were most commonly members of Equestrian Sports New Zealand, Harness or Thoroughbred Racing associations. This finding provides insight into existing networks and pathways that could be conduits for the dissemination of knowledge and information intended to address the human factors of horse injury during road transport and other topics of importance to horse health and welfare. Fewer respondents were associated with the New Zealand Pony club compared with other industry organizations (Table 2), presumably because the human ethics based criteria for participation was being older than 16 years old, excluding the more than 80% of members that are younger than 16 years (30). However, the majority of our respondents reported holding a New Zealand Pony Club certificate suggesting that the country's largest equestrian association plays and important role in the education of young people in aspects of horsemanship (30, 32). This organization may therefore be an important conduit for the education of less experienced people in the safe road transport of horses. It is noteworthy that although moving horses very frequently (80% of respondents moving horses from daily to fortnightly), almost 40and 20% of our respondents, respectively did not have any equine industry related qualifications or organizational memberships.

The survey required respondents to self-assess their ability to identify a horse in distress, and the likelihood of them assessing the fitness of their horse prior to transport, as required by the AWC. However, only one in five indicated that they had a high understanding of the AWC. The lack of knowledge of the AWC was reflected in the one out of three respondents that did not report assessing the fitness of their horse for transport. This practice is mandatory in the New Zealand Animal Welfare Code, and such evaluations reduce the risk of transport related health problems (12). It is notable that the AWC itself does not clearly define the terms “distress” or “fitness,” or give guidelines on how to make these determinations. Similarly these terms were not defined in the survey questions, and therefore imprecision with respect to the definition of this variable requires cautious interpretation of this finding. Defining such terms may improve the ability of horse owners to comply with the code, and the quality of the information obtained from surveys such as the one described here.

The published incidence of transport related injuries differs between performance and slaughter horses, due to differences in perceived market value, modes of transport and commercial drivers vs. owner/drivers (11, 33). However, this study focused only on racing, equestrian sports performance and pleasure horses transported by road in New Zealand, rather than those transported to slaughter. The percentage of respondents reporting a traumatic injury associated with road transport in New Zealand was lower than that found in two contemporary Australian surveys (14, 34). This discrepancy may be associated with differences in survey methodology, characteristics of the respondents, the number of horses represented, type of journey and period of investigation. In the current survey, respondents were usually older, more experienced, and with fewer horses in their care than found in a comparable Australian survey (34). Whilst median of age and experience of respondents and number of horses in care were similar between the current survey and that of Riley et al. (14), the latter study collated information on incidents that occurred over a longer period (i.e., 15 years).

Loading and unloading have been considered the phases of transport during which horse are at greatest risk of injury (15, 35) and have therefore been a focus of other researchers (13). In contrast to other studies (15, 34), the current survey respondents described the majority of injuries as occurring in transit. This higher incidence of injuries during vehicle motion may be related to the journey characteristics including those associated with geographic features of the New Zealand terrain.

In agreement with the literature horse behavior was most frequently perceived by respondents as the suspected cause of injury (14). In a parallel survey of transport related problem behaviors described by the same group of respondents, the authors recently reported that training methods, training aids, and the offering food while traveling are associated with such behaviors (36). Loading and traveling problem behaviors have been identified as risk factors for transport related injuries increasing their odds 3-fold (37). Appropriate training to habituate horses for loading and traveling has been recommended to reduce transport related problem behaviors and consequent injuries (38). In this survey, 80% of the respondents reported training their horses for loading and traveling; this percentage is higher than that reported in Australia (34) and may have contributed to the overall lower incidence of loading injuries. A driving error was mentioned as cause of injury only by 10% of the respondents, higher than that reported in South Australia (14). Nevertheless, this may represent an underestimation by the survey respondents of the importance of driving skills and driving behavior that have been shown as crucial for the balance of the transported animals (39).

The age of the horse handler was a significant risk factor for transport related injury in Australia (12), with horses managed by people younger than 30 years having 4 times the likelihood of reporting an injury in comparison to those managed by people older than 60 years. The New Zealand data support this finding, but additionally have further characterized less experience in horse handling as a human factor. The New Zealand AWC (24) recommends that the competence of those responsible for horses during transport should be demonstrated through practical experience, but does not require any certification or verification. The Australian Code for transport is more specific in that it is strongly recommended that horses should be moved only by people with at least 5 years of driving experience. Our findings support this recommendation, as they confirm that at least 5 years of experience in horse handling is required to reduce the odds of reporting a transport related injury. The odds of an injury were also found to be increased in case of respondents that had only a learner or restricted drivers license (i.e., a no full driving license). Although these drivers require supervision at all times and during the night, respectively, there are no conditions in New Zealand that preclude them from towing or driving a vehicle (with a gross laden weight < 4,500 kg) containing livestock. To the authors knowledge this is the first study to demonstrate an association between horse transport injury with driver qualifications as a de facto variable for driver experience. A review of age related safety in professional heavy vehicle drivers has demonstrated that drivers >27 years old (i.e., less experienced) are more likely to be involved in an accident or fatality (40). Similar findings have been described for drivers that transport cattle (16).

The association between having reported a transport related injury and respondents who did not always check the vehicle before transport in the current study is in agreement with previous work that found a similar trend (14). However, it is unclear if respondents who transported horses in floats interpreted the question about following ‘a mechanical checklist on the transportation vehicle' in relation both the tow vehicle and the float. An appropriate check list of the vehicle has been recommended as a best practice (41) because often the accidents involving horse trailers has been associated with mechanical failure of the transport vehicle (14).

This survey explored the possible connection between injury and professional or amateur industry involvement, as well as, the horse related activity or sport in which respondents were engaged. Professional respondents (people that were involved with horses for business purposes or as part of their employment) had higher odds of reporting an injury. Although the current survey was not able to quantify levels of exposure, this finding may reflect the higher rates of movement reported for commercial properties and activities (20, 21) compared to those reported for non-commercial properties in New Zealand (22). In Europe formal certification is mandatory for all drivers, transporters and people moving horses for commercial purposes or for distances >60 km in the case of non-commercial road movement (42). If a similar process was introduced into New Zealand, interpretation of the data in this report suggests that certification may be relevant also for drivers traveling <60 km.

The spectrum of equine related activities within New Zealand is diverse (20, 21). In the current study population eventing was associated with higher odds of reporting a transport injury in comparison with recreational riding. Other equestrian sports disciplines also had increased odds of injury, but their 95% confidence intervals overlapped with unity. Eventing is considered a sport that places both horses and riders at a higher risk of injury than many other equestrian activities (6), and is the most physically demanding of the Olympic equestrian sports (43, 44). Singer et al. (44) reported that the majority of the injuries to eventers in training and competitions involve superficial trauma in the form of contusions, abrasions or full thickness skin wound. These injuries often became apparent within the 24 h of the performance activity, and the incidence of injuries is considered as underestimated because many horses travel home soon after an event. It is unknown if eventing horses described in the current survey were returning from performance events, or if this may have contributed to respondents reporting transport related injury in the current survey. Future studies should seek to elicit this information (37).

In the final multivariate model, less experience in horse handling, being involved in eventing, and being a professional provided the greatest contributions to modeling the risk of respondents reporting an injury. Professionals are usually regarded as having more experience in horse handling, but it was reported that they did not have a high level of understanding and application of learning theory (45). Moreover, as discussed above, their exposure to transport injury is higher based on greater numbers of journeys per year and a greater likelihood of having more horses in their care (12, 20, 29, 34). As alluded to previously, those people less experienced with horse handling may benefit from structured educational programs and a qualification framework that assists and accelerates the acquisition of the necessary best practice skills for the safe transport of horses (41, 46). Our data suggest that this may be particularly important not only for those professionally involved in the equine industry, but also for those with <5 years of experience in horse handling in order to mitigate the human factors contribution to the risk of equine transport related injury.

Studies of this type and design reported here share a number of limitations common to survey-based studies (25, 26) that must be considered in interpreting the results. The surveys were not distributed randomly as there is no national database in New Zealand that identifies the location of horse owners that could be used to facilitate such an approach. Based upon the knowledge of the authors of the New Zealand equine industry, respondents were invited to fill the survey to report their equine industry background and transport related-injury. Online survey distribution selects for respondents with internet access, but awareness of the survey may have been increased via other means following the initial telephone contacts with industry leaders and administrators. Selection bias may also occur by people self-selecting to participate in the survey if they are more highly experienced in transport, or have past experiences of transport related injuries. Women replied more than men to the survey, and this may reflect the higher participation of women in online surveys observed in previous studies (34, 47) and/or the greater participation of this gender in horse-related activities (17, 48). The age demographics of the study population in the current report suggest that both more and less experienced respondents participated. It should nevertheless be acknowledged that prevalence of injury may have been under- or overestimated due to the afore mentioned selection bias. Transport-related injuries were identified by participant recollections of past events, and therefore data are vulnerable to recall bias. The authors attempted to reduce the likelihood of this bias by limiting the survey to recollection of only the previous 2 years. The researchers also attempted to randomize the injury on which participants reported, by instructing participants to respond in relation to the last horse injury only (not the most serious injury). A further limitation is the reliance upon respondents to self-assess injury in their horses. Others have used this approach to the study of equine health (49), but significant differences between owner (under) reported health conditions and veterinary assessment have been recognized (50). It should also be remembered that surveys of this type may provide information on the association between risk factors and injury, but do not provide evidence of causation. Therefore, those human factors found to be associated with the risk of injury require further investigation, preferably prospective, to more confidently identify their true significance with respect to transport related injury.

Although strenuous efforts were made to recruit respondents across all sectors of the New Zealand equine industry (numbers of respondents from each sector are reported), it is possible that there are differences in the representativeness of the survey results between sectors (sampling bias). Notwithstanding these limitations, this study has generated important insights into demographics of New Zealand equine industry members and their relationship to the road transport of horses and the risk of injury. The findings from the current study may assist in supporting improvements in the AWC with respect to road transport and encourage its more widespread utilization. They may also inform the design of prospective studies aimed at elucidating causation, so as to reduce the incidence of traumatic injuries and safeguard horse welfare. Educational strategies that ensure the application of learning theory in horse handling and use of best practices for the road transport of horses by amateur and professional industry respondents should be developed and implemented to enhance horse welfare and reduce arising financial losses.

## Conclusions

Several human factors associated with transport related injuries of horses in New Zealand were identified. Overall, the experience in horse handling of the people moving a horse by road and their professional involvement with the horse and the equine industry seem to be the most important human factors associated with transport related injuries in horses. Further prospective studies are needed to confirm our preliminary findings.

## Author contributions

BP, DG, and CBR contributed conception and design of the study. DG and CBR organized the database. BP performed the statistical analysis and wrote the first draft of the manuscript; CWR, KT, and CBR wrote sections of the manuscript. All authors contributed to manuscript revision, read and approved the submitted version.

### Conflict of interest statement

The authors declare that the research was conducted in the absence of any commercial or financial relationships that could be construed as a potential conflict of interest.
