# Supplementary material for: A Survey-Based Investigation of Human Factors Associated With Transport Related Injuries in Horses
Source: Front Vet Sci. 2018 Nov 22;5:294. doi: 10.3389/fvets.2018.00294 (PMC6262080; doi:10.3389/fvets.2018.00294)
Supplement: Supplementary file 1 [file Table_1.DOCX]

Supplementary Material

Human factors contributing to the risk of equine road transport injury in New Zealand

Barbara Padalino*, Chris W Rogers, Danielle Guiver, Kirrilly R Thompson, Christopher B Riley

*** Correspondence:** Dr. Barbara Padalino; barbara.padalino@cityu.edu.hk

# Supplementary Data

Supplementary Material should be uploaded separately on submission. Please include any **Survey**

1. What is your sex?

- Male
- Female
- Other

1. What is your age (years)?
2. What is the postcode of the town or place where your horse is kept?
3. What section of the horse industry or sport are you primarily involved in?

- Thoroughbred racing
- Standardbred racing
- Pony club
- Endurance
- Horse breeding
- Recreational riding
- Dressage
- Show jumping
- Eventing
- Reining
- Showing
- Polo
- Other (please specify) ____________________

1. Are you a registered member of one or more of these organisations? (tick all that apply)

- I am not a registered member of any horse related organisation
- New Zealand Thoroughbred Racing
- Harness Racing New Zealand
- Equestrian Sports New Zealand
- New Zealand Pony Club Association
- Other (please specify) ____________________

1. What is your relationship with the horse(s)?

- Mostly amateur - involved with horses as a hobby or for recreational purposes
- Mostly professional - involved with horses as part of my job

1. What do you do professionally with horses?
2. What amateur activities do you do with horses?
3. How many years of experience do you have with handling horses?
4. Do you have any qualifications for the equine industry? (tick all that apply)

- New Zealand Pony club certificate (please specify) ____________________
- Racing (please specify) ____________________
- Equestrian Sports New Zealand qualification (please specify) ____________________
- New Zealand national certificate - horse related (please specify) ____________________
- Other (please specify) ____________________
- No, I do not have any specific horse related qualifications

1. Approximately how many horses are kept where you keep your horse(s)?
2. What type of driving licence do you hold? (tick all that apply)

- Learners
- Restricted
- Full
- Heavy vehicle licence

1. How do you rank your understanding of the Animal Welfare Code (Transport within New Zealand, 2011)?

- 1 – low
- 2 – some
- 3 - moderate
- 4 - high
- 5 - very high

1. How do you grade your ability in identifying a horse in distress?

- 1 - low
- 2 - some
- 3 – moderate
- 4 – high
- 5 - very high

1. How often do you transport your horse(s)?

- Daily
- 2 to 5 times a week
- Once weekly
- Fortnightly
- Monthly
- Less than once a month

1. What is the average kilometers traveled per trip when you have transported your horse(s) in the last year?
2. How likely are you to assess the fitness of your horse(s) for travel before transporting it?

- 1 - never
- 2 – sometimes
- 3 - about half the time
- 4 - most of the time
- 5 - always

1. How likely are you to follow a mechanical check list on the transportation vehicle before traveling?

- 1 – never
- 2 - sometimes
- 3 - about half the time
- 4 - most of the time
- 5 - always

1. Have you specifically trained your horse(s) for loading and traveling?

- No
- Yes

1. Has your horse(s) had any injuries related to transportation within the last 2 years?

- No
- Yes

1. Approximately when did the most recent transport related injury to the horse occur (month and year)?
2. When during the trip did the injury occur?

- Pre- loading
- Loading
- Travelling
- Unloading
- I don't know

1. How do you think the injury occurred? (tick all that apply

- A mistake by the driver
- A problem with the horse
- Poor road conditions
- Mechanical problem with the transport vehicle
- Vehicle collision
- Other (please specify) ____________________

1. What type of injury occurred? (tick all that apply)

- Shallow cut or wound
- Deep cut or wound
- Fracture or broken bone
- Bruise
- Skin or tail rubbed raw
- Other (please specify) ____________________

1. What was your involvement with the transportation of the injured horse? (tick all that apply)

- Driver
- Passenger
- Person loading the horse
- Person unloading the horse

Supplementary Tables

**Table S1. Distribution pathways used for the survey**

| **Category** | **Name** | **Method** |
| --- | --- | --- |
| Equine organisation | Manawatu Pony Club  <http://www.sporty.co.nz/manawatupc> | Initial contact with Executive committee member by telephone. Information brochure and survey link then emailed for distribution via email, web and Facebook pages |
|  | Clydesdale Horse Society of New Zealand  <http://www.clydesdale.org.nz/> | Information brochure and survey link emailed to President and Secretary for distribution via email |
|  | New Zealand Riding Clubs & Bridleways  <https://nzridingclubsbridleways.nz/> | Initial personal contact with President and Committee member, and then by telephone. Information brochure and survey link then emailed for distribution via email, web and Facebook pages |
|  | New Zealand Carriage Driving Society  <https://www.carriagedrivingnz.co.nz/> | Initial contact with Treasurer by telephone. Information brochure and survey link then emailed for distribution via email, web and Facebook pages |
|  | Equestrian Sports New Zealand Endurance  <http://www.nzequestrian.org.nz/endurance/> | Initial personal contact with Board member and ordinary member. Information brochure and survey link then emailed for distribution via email, web and Facebook pages |
|  | New Zealand Racing & New Zealand Thoroughbred breeders  <https://www.nzracing.co.nz/Home.aspx>  <https://www.nzthoroughbred.co.nz/> | Initial contact with Keeper of Studbook and Registration Manger and General Manager. Information brochure and survey link then emailed for distribution via email, web and Facebook pages |
| Publication | Horse and Pony Magazine  <https://horseandponymag.com/> | Initial contact with editor by telephone and email. Information brochure and survey link then emailed for distribution |
| Equine business | Paul Douglas Floats, Pahiatua, New Zealand  <https://www.pauldouglasfloats.com/> | Initial contact with owner by telephone. Information brochure and survey link then emailed for distribution via email, business web and Facebook pages |
|  | Tielcey Park, Palmerston North  [www.tielceyparkstables.co.nz/](http://www.tielceyparkstables.co.nz/) | Initial contact with owner by telephone. Information brochure and survey link then emailed for distribution via email, business web and Facebook pages |
| Institution | Institute of Veterinary, Animal and Biomedical Sciences, Massey University, New Zealand  [www.massey.ac.nz/](http://www.massey.ac.nz/) | Information brochure and survey link emailed to list serve group |

**Table S2:** **Wald test’s P values generated from univariate regression analysis**

| Predictive Variable | Injury |
| --- | --- |
| Gender | 0.265 |
| Age | <0.001 |
| Sector | 0.019 |
| Involvement | 0.107 |
| Experience | 0.005 |
| Qualification | 0.415 |
| Driving Licence | 0.045 |
| AWC | 0.300 |
| Distress | 0.615 |
| Assessment of fitness for transport | 0.144 |
| Mechanical Checklist | 0.048 |
| Horse training | 0.514 |
| Number of horses | 0.379 |
| Journey frequency | 0.514 |
| Journey duration | 0.331 |

**Table S3: Univariate of the variables which were included in the multivariate model**

| **Variable** | **Category** | **No Injuries**  **n(%)** | **Injuries n(%)** | **OR** | **95%CI** | **P ^a^** |
| --- | --- | --- | --- | --- | --- | --- |
| Involvement | Amateur | 709 (62.6) | 142 (12.5) | Ref |  | 0.107 |
|  | Professional | 223 (19.7) | 59 (5.2) | 1.32 | 0.94-1.85 |  |
| Assessment of fitness for transport | 5 -always | 615 (54.5) | 116 (10.3) | Ref |  | 0.144 |
|  | 4- most of the time | 206 (18.2) | 57 (5.0) | 1.46 | 1.03-2.08 |  |
|  | 3- about half the time | 41 (3.6) | 9 (0.8) | 1.16 | 0.55-2.45 |  |
|  | 2- sometimes | 53 (4.7) | 15 (1.3) | 1.50 | 0.81-2.75 |  |
|  | 1- never | 16 (1.4) | 1 (0.1) | 0.33 | 0.43-2.51 |  |

**
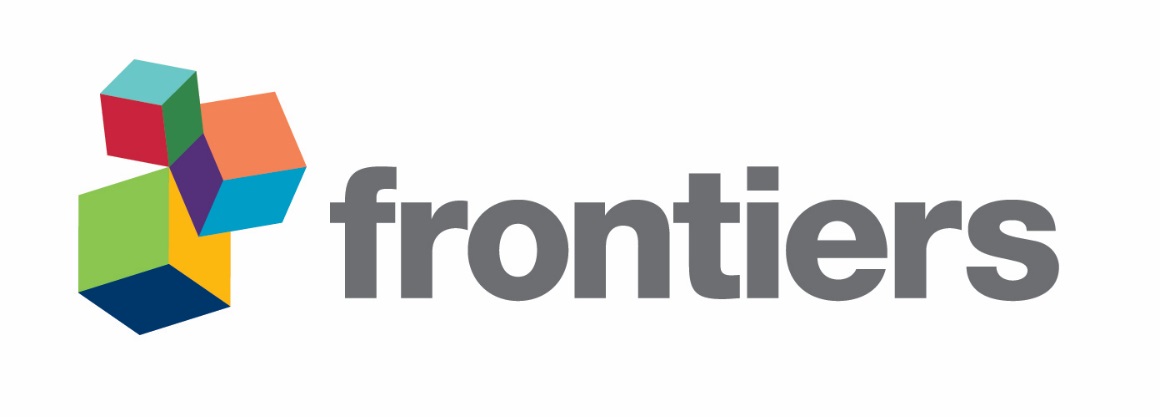
**
